# Seasonal patterns of dengue fever and associated climate factors in 4 provinces in Vietnam from 1994 to 2013

**DOI:** 10.1186/s12879-017-2326-8

**Published:** 2017-03-20

**Authors:** Hu Suk Lee, Hung Nguyen-Viet, Vu Sinh Nam, Mihye Lee, Sungho Won, Phuc Pham Duc, Delia Grace

**Affiliations:** 1 International Livestock Research Institute, Regional Office for East and Southeast Asia, Room 301-302, B1 Building, Van Phuc Diplomatic Compound, 298 Kim Ma Street, Ba Dinh District, Hanoi, Vietnam; 2 Vector Borne Diseases and Training, National Institute of Hygiene and Epidemiology, Hanoi, Vietnam; 3 Medical microbiology Department, The Royal Bournemouth Hospital, Bournemouth, UK; 4 Graduate School of Public Health, Seoul National University, Seoul, Korea; 5 Interdisciplinary Program of Bioinformatics, Seoul National University, Seoul, Korea; 6 Institute of Health and Environment, Seoul National University, Seoul, Korea; 7 Center for Public Health and Ecosystem Research (CENPHER), Hanoi School of Public Health, Hanoi, Vietnam; 8 International Livestock Research Institute, Nairobi, Kenya

**Keywords:** Vietnam, Dengue fever, Monthly incidence rate, Seasonality, Climate factors, Temperature and precipitation

## Abstract

**Background:**

In Vietnam, dengue fever (DF) is still a leading cause of hospitalization. The main objective of this study was to evaluate the seasonality and association with climate factors (temperature and precipitation) on the incidences of DF in four provinces where the highest incidence rates were observed from 1994 to 2013 in Vietnam.

**Methods:**

Incidence rates (per 100,000) were calculated on a monthly basis from during the study period. The seasonal-decomposition procedure based on loess (STL) was used in order to assess the trend and seasonality of DF. In addition, a seasonal cycle subseries (SCS) plot and univariate negative binomial regression (NBR) model were used to evaluate the monthly variability with statistical analysis. Lastly, a generalized estimating equation (GEE) was used to assess the relationship between monthly incidence rates and weather factors (temperature and precipitation).

**Results:**

We found that increased incidence rates were observed in the second half of each year (from May through December) which is the rainy season in each province. In Hanoi, the final model showed that 1 °C rise of temperature corresponded to an increase of 13% in the monthly incidence rate of DF. In Khanh Hoa, the final model displayed that 1 °C increase in temperature corresponded to an increase of 17% while 100 mm increase in precipitation corresponded to an increase of 11% of DF incidence rate. For Ho Chi Minh City, none of variables were significant in the model. In An Giang, the final model showed that 100 mm increase of precipitation in the preceding and same months corresponded to an increase of 30% and 22% of DF incidence rate.

**Conclusion:**

Our findings provide insight into understanding the seasonal pattern and associated climate risk factors.

## Background

Dengue fever (DF) is a mosquito-borne viral disease which is a leading cause of illness and death in tropical and subtropical countries [1, 2]. Currently, four dengue virus serotypes (DEN-1, DEN-2, DEN-3 and DEN-4) are circulating in Asia, Africa and the America [1–3]. Infection with one of those serotypes does not have cross-protective immunity, so people in endemic areas can have four serotypically different dengue infections during their lifetimes [4]. It was estimated that 3.5 billion people are living in areas at risk for infection with approximately 390 million dengue infections annually (95% credible interval 284–528 million), of which 96 million (67-136 million) clinically manifest [5, 6]. It becomes a major public health problem due to expanding geographical distribution with climate change and an evolution from epidemic cycle with long term intervals to endemic with seasonal patterns [7–11]. Climate variability/changes have a significant impact on vector populations. Factors, such as temperature, precipitation and humidity can influence vector development/survival rates, behavior and habitats [12–14]. Previous studies have suggested that temperature and precipitation were associated with the occurrence of DF, but relationships have not been consistently described [15–19]. For instance, in Thailand, temperature and precipitation were positively associated with dengue transmission [20]. In a Taiwanese study, the incidence rate was negatively associated with monthly temperature and relative humidity [18]. These discrepancies may be attributed to different climate/environmental conditions and different data analysis methods.

In Vietnam, a National Dengue Control Programme (NDCP) was established in 1999, which relies mainly on vector control to decrease the transmission from vectors to humans. However, dengue is still a leading causes of hospitalization, and Vietnam has the highest number of cases in the Western Pacific region [21]. To our knowledge, few studies have been conducted to assess the seasonal pattern of DF and its associated climate factors in Vietnam. The main objective of this study was to evaluate the seasonality and association with climate factors (temperature and precipitation) on the incidences of DF in Hanoi, Khanh Hoa, Ho Chi Minh City and An Giang provinces where the highest incidence rates were observed from 1994 to 2013 in Vietnam.

## Methods

### Study locations and data

**Fig. 1:**
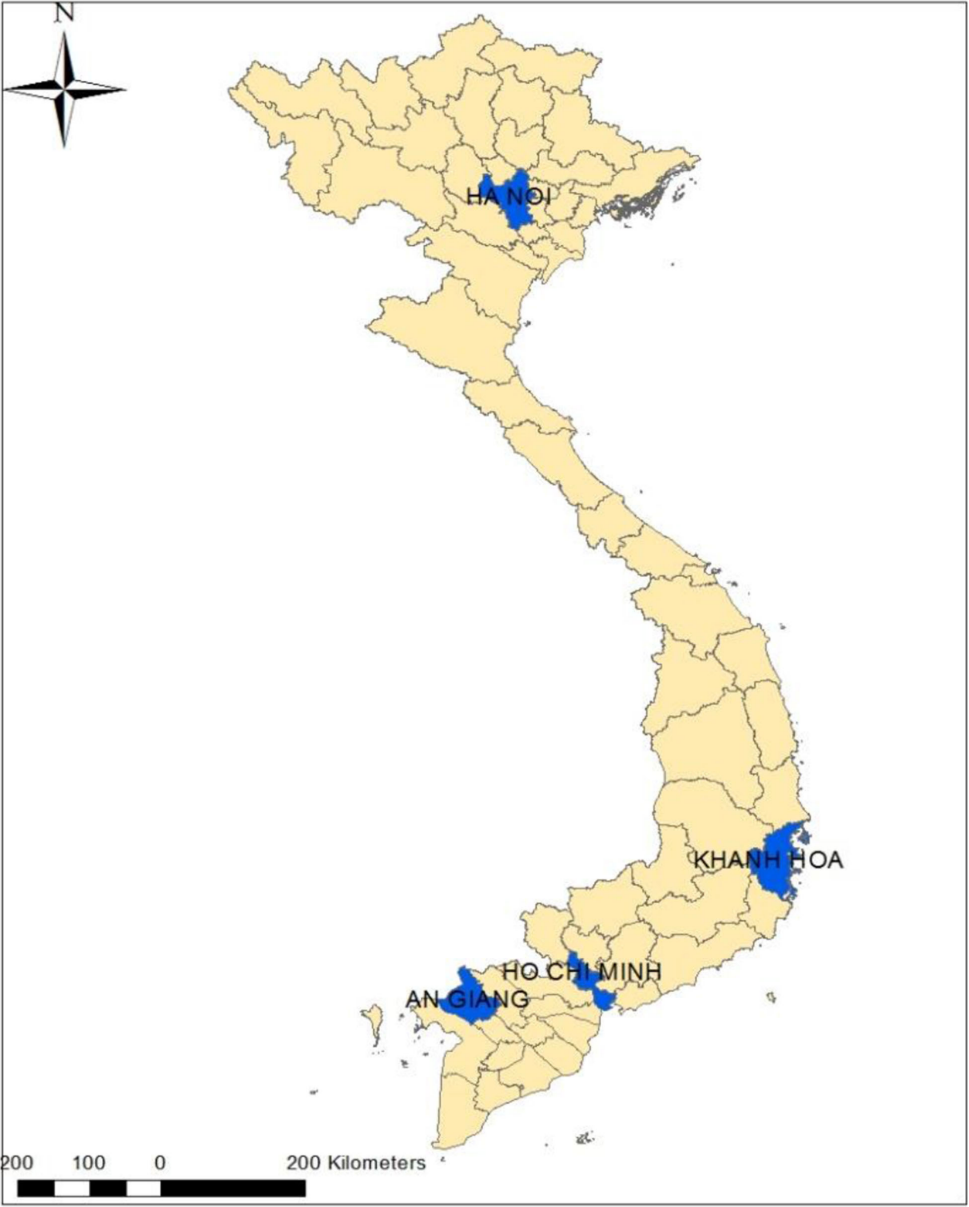
Map of the area in Hanoi, Khanh Hoa, Ho Chi Minh City, An Giang provinces

Hanoi (population: 6.7 million), Khanh Hoa (population: 1.2 million), Ho Chi Min City (population: 7.5 million) and An Giang (population: 2.2 million) provinces with the highest incidence rates were selected for this study, which are located in North, Central and Southern part of Vietnam, respectively (Fig. 1). The annual temperature ranges from 16 °C to 29 °C in Hanoi whereas other three provinces are relatively higher between 24 °C and 29 °C (Table 1). Generally, there are two seasons: the rainy (from May to October) and dry seasons (from November to April) in Vietnam.

**Table 1:** Monthly weather conditions in Hanoi, Khanh Hoa, Ho Chi Minh City and An Giang from 1994 to 2013

Month	Hanoi		Khanh Hoa		Ho Chi Minh City		An Giang	
	Temperature (°C)	Precipitation (mm)	Temperature (°C)	Precipitation (mm)	Temperature (°C)	Precipitation (mm)	Temperature (°C)	Precipitation (mm)
Jan	16.67	19.59	24.17	49.74	27.18	104.39	25.79	8.97
Feb	18.36	21.9	24.81	13.87	26.77	156.74	26.32	4.97
Mar	20.62	47.98	26.04	33.02	27.59	147.74	27.58	15.04
Apr	24.44	68.95	27.70	52.95	27.06	138.36	28.78	84.02
May	27.34	205.05	28.55	101.34	27.15	124.34	28.43	147.42
Jun	29.34	246.82	28.97	56.61	27.45	136.61	27.95	114.16
Jul	29.25	307.14	28.74	53.57	27.19	110.26	27.41	175.45
Aug	28.51	316.37	28.69	52.94	27.23	131.81	27.73	215.41
Sep	27.43	203.24	27.96	201.51	27.09	161.86	27.62	160.4
Oct	25.52	160.29	26.89	359.30	27.46	118.08	27.52	252.95
Nov	22.18	80.54	25.98	429.06	27.20	145.00	27.36	155.66
Dec	18.38	39.13	24.76	198.70	27.61	142.75	26.22	48.96
Summary^a^	24.0 ± 4.6	143.08 ± 131.07	26.94 ± 1.76	133.55 ± 175.69	27.24 ± 0.96	134.82 ± 116.32	27.39 ± 1.17	115.28 ± 113.58

^a^mean and standard deviation

According to the national surveillance system of infectious diseases in Vietnam, DF is one of the 28 pathogens which are reported on a monthly basis by the preventive medicine networks. A case of dengue is determined as a suspected case who meets the WHO (World Health Organization) case definition: acute febrile illness (≥38 °C) of 2-7 days duration with two of the following symptoms: severe headache, retro-orbital pain, nausea, vomiting, myalgia, arthralgia, hemorrhagic manifestations and leucopenia [22]. Most of cases are clinically diagnosed and only 10-20% of cases are serologically confirmed. The numbers of cases and deaths are collected at provincial preventive medicine centers which are reported to the regional preventive medicine institutes, and then to the National Institute of Hygiene and Epidemiology (NIHE). Annually, the NIHE and then Ministry of Health (MOH) publish books of reported dengue fever cases. We obtained the data from the annual book of communicable diseases published from 1994 and 2013 of DF cases, these were entered in Excel sheets by our research team. In addition, monthly meteorological data [total precipitation (100 mm) and average temperature (°C)] were obtained from four provinces (source: Institute of Meteorology and Hydrology and Climate Change).

### Data analysis

Incidence rates (per 100,000) were calculated on a monthly basis during the study period. The seasonal-decomposition procedure based on loess (STL) was used in order to assess the trend and seasonality of DF. This is a statistical technique to decompose a time series dataset into trend, seasonal and remainder on a yearly basis (12 months) [23]. In addition, a seasonal cycle subseries (SCS) plot and univariate negative binomial regression (NBR) model were used to evaluate the monthly variability with statistical analysis [24]. The SCS displays a horizontal line for the average incidence rate of each month over the total period, and each vertical line above and below the horizontal line indicates the specific incidence rate for that month in each year of the data. In order to evaluate the monthly differences, univariate NBR models were constructed for each province while February was used as reference.

A generalized estimating equation (GEE) was used to assess the relationship between monthly incidence rates and weather factors (temperature and precipitation), because serial correlations were detected between observations. We employed the GEE with Autoregressive (AR)(1) as working correlation matrix and sandwich estimators were used to adjust the residual correlations. It should be noted that sandwich estimator in GEE guarantees the robustness against the misspecified correlation structure [25]. A negative binomial distribution (NBD) was selected for the GEE model which includes a Poisson distribution with an extra-dispersion term (alpha (α)). Generally, a Poisson regression distribution should be considered for count data but the Poisson distribution model is not able to take into account the overdispersed count outcomes [26, 27]. A likelihood ratio was implemented in order to confirm the presence of overdispersion, and the test showed the presence of overdispersion in the model.

For the variable screening, we conducted the correlations between monthly temperature and precipitation while the correlations between the original monthly variables and their one lag (preceding month) counterparts were investigated in order to evaluate the possible lagged effect in the model. The linearity of effect of temperature and precipitation on DF incidence was explored using loess smoothing curves. If there was evidence of non-linearity, a quadratic function of the predictor was considered in the model if *P* < 0.05. For the model building, the strong correlation (*r* = 0.72, *P* < 0.001) was observed between temperature and precipitation for Hanoi, therefore we developed two multivariable models – a model had the temperature without precipitation and vice versa. For other provinces, both variables (temperature and precipitation) with one lag were screened in the models.

Variables with *P*-values <0.05 were considered to be significant in the final models. In addition, the Bonferroni-adjusted significance level obtained by dividing the significance level α, by the number of *P*-values was used for multiple comparison problems. The accuracy of the proposed model was evaluated with the root-mean-square error (RMSE). To minimize the overfitting problem, our data were split into train and test data. Train data consist of observations from January 1995 to December 2012, and were used to provide the linear model. The remaining data (from January 2013 to December 2013) were assigned as test data, and used to calculate RMSE. The results for the NBR were demonstrated as incidence rate ratio (IRR) and 95% confidence interval (CI). All data were created in Microsoft Excel 2010 and analyzed using R (version 3.2.2) and STATA (version 14.0, Stata Corp, College Station, TX, USA). ArcGIS version 10.3 ArcMap (ESRI, Redlands, CA, USA) was used to generate the map. This study was approved by the Hanoi Medical University Institutional Review Board (HMU IRB: no. 00003121), Vietnam.

## Results

### STL analysis and seasonal cycle subseries by province

**Fig. 2:**
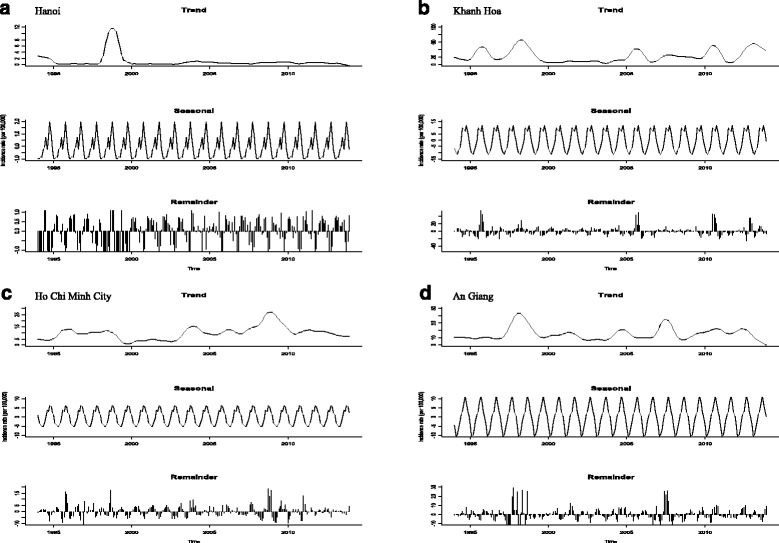
Seasonal-trend decomposition (Trend, Seasonal and Remainder) of the monthly incidence rates of DF in four provinces from 1994 to 2013 (Each plot has different Y-axis scales)

**Fig. 3:**
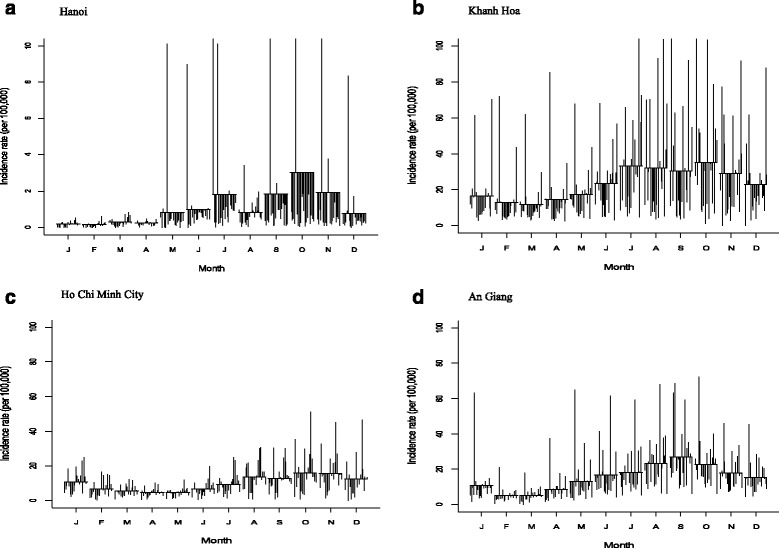
Seasonal cycle subseries plot of the monthly incidence rates of DF in four provinces from 1994 to 2013 (only Hanoi has different Y-axis scales)

In Hanoi, a total of 46,043 cases (average 3.436 per 100,000; 95% CI: 3.432-3.439) were reported from 1994 to 2013. The trend plot showed the mild fluctuations (apart from dramatically increase and decrease incidence in 1998) (Fig. 2-a: first plot). The STL plot exhibited the seasonal patterns with a strong peak during the last half of the calendar year and a smaller peak in July (Fig. 2-a: second plot). The remainder component showed varying residuals with intermittently large values. The seasonal cycle subseries plot demonstrated that the highest incidence rate was noted in October whereas the lowest was in February (Fig. 3-a). An univariate NBR model showed that all months (except for March and April) were at increased risk for DF incidence rates compared to February at an alpha level of 0.05 while January was not statistically significant with Bonferroni adjustment (*P* < 0.05/11) (Table 2). In particular, incidence rates of disease were highly increased in October followed by November and September.

**Table 2:** Univariate negative binomial regression (NBR) models for the DF incidence rates by month with incidence rate ratio (IRR) and 95% confidence interval (CI)

Month	Hanoi	Khanh Hoa	Ho Chi Minh City	An Giang
January	2.73 (1.19-6.26)*	1.26 (0.73-2.18)	1.61 (1.03-2.53)*	2.12 (1.39-3.22)*†
February	Reference: 1	Reference: 1	Reference: 1	Reference: 1
March	1.36 (0.59-3.14)	0.90 (0.52-1.56)	0.84 (0.54-1.32)	1.04 (0.68-1.59)
April	1.43 (0.62-3.30)	1.12 (0.65-1.92)	0.71 (0.45-1.11)	1.69 (1.11-2.56)*
May	5.14 (2.24-11.77)*†	1.34 (0.78-2.30)*	0.71 (0.45-0.11)	2.58 (1.70-3.92)*†
June	6.63 (2.90-15.19)*†	1.79 (1.04-3.09)*	0.99 (0.63-1.54)	3.28 (2.16-4.98)*†
July	14.94 (6.52-34.17)*†	2.55 (1.43-4.39)*†	1.40 (0.89-2.19)	3.58 (2.36-5.45)*†
August	22.02 (9.63-50.34)*†	2.46 (1.43-4.24)*†	2.06 (1.31-3.22)*†	4.57 (3.01-6.94)*†
September	38.47 (16.83-87.94)*†	2.35 (1.36-4.04)*†	1.91 (1.22-2.99)*	5.26 (3.46-8.00)*†
October	55.07 (23.65-123.59)*†	2.69 (1.56-4.63)*†	2.40 (1.53-3.76)*†	4.49 (2.95-6.83)*†
November	46.69 (20.43-106.73)*†	2.22 (1.29-3.83)*†	2.35 (1.50-3.67)*†	3.49 (2.30-5.31)*†
December	13.44 (5.88-30.74)*†	1.75 (1.02-3.01)*	1.84 (1.18-2.89)*†	3.01 (1.98-4.58)*†

* = statistically significant (*P* < 0.05)

† = Bonferroni adjustment (*P* < 0.05/11)

**Table 3:** Final NBR models of DF incidence rates in Hanoi, Khanh Hoa, Ho Chi Minh City and An Giang

Province/Variable	Adjusted incidence rate ratios (IRRs)	95% CI	*P* value	RMSE
Hanoi (0.717, <0.001)				
Monthly average temperature (°C)	1.13	1.09-1.17	<0.001^a^	14.92
Khanh Hoa (−0.140)				
Monthly average temperature (°C)	1.17	1.09-1.26	<0.001^a^	22.40
Monthly total precipitation (100 mm)	1.11	1.03-1.20	0.004^a^	
Ho Chi Minh City (0.232)				
Null	Null	Null	Null	Null
An Giang (0.1672)				
Monthly total precipitation (100 mm) in the preceding month	1.30	1.13-1.50	<0.001^a^	44.06
Monthly total precipitation (100 mm) in the same month	1.22	1.06-1.40	0.006^a^	

^a^ = Bonferroni adjustment (*P* < 0.05/4)

In Khanh Hoa, a total of 61,404 cases (average 23.473 per 100,000; 95% CI: 23.468-23.479) were recorded during the study period. The trend plot showed the large fluctuations with an apparent overall increase and decrease in 1995, 1998, 2005, 2010 and 2012 (Fig. 2-b: first plot) while the STL plot had two peaks with seasonal pattern in the second half of each year (Fig. 2-b: second plot). In the remainder component, large residuals were observed in 1996, 2005 and 2010. The seasonal cycle subseries showed that incidence rates were relatively higher in the second half (Fig. 3-b). In an univariate NRB analysis, there were significantly increased incidences of disease from May to December compared to February (α = 0.05) while May, June and December were not statistically at an increased risk of acquiring the disease when Bonferroni correction was applied (Table 2).

For Ho Chi Minh City, a total of 61,404 cases (average 10.349 per 100,000; 95% CI: 10.348-10.351) were reported during the study period. The STL trend plot presented the largest fluctuations with its peaks in 1997, 2003 and 2008 (Fig. 2-c: first plot). The seasonal plot had primary and secondary peaks at the end of year (Fig. 2-c: first plot). In the remainder plot, large values were observed in 1995, 1998, 2008 and 2011. The highest rates on the seasonal cycle subseries plot were in October, November and August whereas the lowest rates were in May, April and March (Fig. 3-b). There were significantly increased incidence rates of disease in January and from August to December compared to February whereas the risk was significantly reduced between March and June at an alpha level of 0.05. For the Bonferroni adjustment, September was not statistically at an increased risk.

In An Giang, a total of 61,404 cases (average 15.391 per 100,000; 95% CI: 15.381-15.402) were reported during the study period. The STL plot showed the largest fluctuations with its peaks in 1997, 2004, 2009 and 2012 (Fig. 2-d: first plot). The seasonal plot showed a single peak in the early second half of each year (Fig. 2-d: second plot). The remainder plot appeared to have random variations although large values were noted in 1998 and 2007. The highest rates on seasonal cycle subseries plot were in September, October and August whereas the lowest rates were observed in March, April and March (Fig. 3-d). In an univariate NBR analysis, there were significantly increased risks of disease in all months (except for March) with α = 0.05 while March and April were statistically not at an increased risk with Bonferroni correction.

### GEE model by each province

In Hanoi, the final model showed that 1 °C rise of temperature corresponded to an increase of 13% in the monthly incidence rate of DF (Table 3) while the precipitation was not significant in the model. The preceding month for temperature was not significant in the final model while linearity was observed between temperature and DF incidence rate with RMSE = 14.92.

In Khanh Hoa, the final model displayed that 1 °C increase in temperature corresponded to an increase of 17% while 100 mm increase in precipitation corresponded to an increase of 11% of DF incidence rate with RMSE = 22.40. The preceding months for both variables were not statistically significant in the models. In addition, linearities were observed. For Ho Chi Minh City, none of the variables were significant in the model. In An Giang, the final model showed that 100 mm increase of precipitation in the preceding and the same months corresponded to an increase of 30% and 22% in the monthly incidence rate of DF with RMSE = 44.06, respectively while linearity was observed (apart from 1 outlier). All variables in the final modes were statistically significant with Bonferroni adjustment (*P*-value < 0.05/4).

## Discussion

The aim of this study was to evaluate the seasonal pattern and association between incidence rates and weather risk factors in four provinces during last 20 years (1994-2013). We found that increased incidence rates were observed in the second half of each year (from May through December) which is the rainy season (“called dengue season”) in Vietnam [28, 29]. The big outbreaks appear to be occurring every 10 years (in 1987, 1998 and 2010). We were able to identify a number of some peaks associated with sudden increase in each province which were assumed to be related to emergence of new serotypes or drop in herd immunity.

Dengue fever occurs across the country, with the highest number of cases in the southern part of the country. In Hanoi (capital city), average incidence rates were lower (3-7 times lower) than other three provinces. However, dramatically high IRRs for the rainy season were observed compared to other provinces. This may be attributed to the more active surveillance, easy access to the healthcare system and increased public awareness (including clinicians and general public). However, an absolute number of suspected cases are high during the rainy season across the country, so that increased awareness is less likely to influence on our results. The National surveillance system for DF has been consistently collecting cases from local authorities in collaboration with well-trained clinicians so that surveillance dataset may be representative of all regions of Vietnam. In terms of clinical diagnosis, results of hematocrit and thrombocyte counts could be used to help to differentiate between dengue and malaria or JE/meningitis. In addition, distributions of malaria, JE or meningitis are different compared to dengue (dengue in urban, sub-urban and populated areas; mostly malaria in mountainous areas and JE in rural areas with rice field). Therefore, doctors also can utilize geographical areas to aid diagnosing patients. We found that temperature and precipitation were positively associated with the occurrence of disease which were consistent with findings from the previous studies [20, 28, 30, 31]. For stance, a positive correlation was observed between rainfall and vector population in Malaysia [32]. In an Indonesian study, it was suggested that higher temperatures had led to shorter incubation periods and increased virus replication [33].

Vietnam has one of the highest rates of urbanization in the world, both spatially and demographically and this is likely to affect the ecology of mosquitoes [34]. One study found that urbanization was positively correlated with density, larval development rate and adult survival rate of vectors, which can potentially increase the transmission of disease [35]. This study highlights the highly dynamic nature of DF epidemiology and the need to base disease surveillance and control on timely and representative data. Moreover, in Vietnam, people have a tendency to store water near to their houses due to a lack of reliable water supply in peri-urban/rural areas. A significant portion of people also live in poorly sanitized areas. The stagnant water and substandard sanitary and hygiene practices can lead to creation of good breeding sites for the vectors. In Ho Chi Minh City, we did not find the association between climate factors and DF. It could be possible that DF incidence is more likely to be influenced by other potential risk factors (such as lifestyle and man-made water containers). Actually, because of various water storage behaviors in different regions, vector population in southern regions is 5-10 times higher than northern regions (unpublished data). For An Giang, DF incidence rate had a significant association with the precipitation in the preceding and current months that may be useful to develop the predictive early warning indicators in that region. Overall, our models have the relatively large RMSEs, therefore in order to improve performance of models, other unmeasured environmental factors (such as control measures, herd immunity demographic/socioeconomic changes and urbanizations) should be considered which may be attributed to dengue transmission.

Several limitations should be addressed as follows. It is possible that DF cases were under reported, especially in rural areas due to lack of healthcare and diagnostic facilities. A retrospective study showed that among acute undifferentiated fever cases, 33.6% of cases were caused by dengue fever, which may suggest the possibility of substantial underestimation in local areas [36]. In addition, there was a possibility of misdiagnosis with other diseases as most of cases were reported based on clinical manifestation and hematocrit/platelet counts. It could be possible that more cases were likely to be diagnosed by clinicians during the rainy season. We were not able to identify the proportions of the 4 different serotypes, or the age and gender of respondents, which could be associated with the likelihood of exposure to the vectors. All four serotypes have been identified in Southern Vietnam [3]. One study found that 18.4% and 2.4% of the patients from Khanh Hoa and Binh Thuan provinces were confirmed with DF virus serotype 4 and 2, respectively [37]. In addition, male cases with ≥15 years of age were predominately reported from 6 Asian countries [38]. This study has suggested that this may be related to gender and age differences due to exposure differences among older adolescents and adults. Further study is necessary in order to identify circulating serotypes and more vulnerable groups depending on the provinces. We assumed that human population was the same on a yearly basis which is not realistic, however, the effect of this was limited because the relatively large denominators could not have an impact on a monthly incidence rate.

The STL and SCS methods have been commonly used in the economic and environmental areas, but have not been commonly used in the epidemiology/medical fields [24, 39, 40]. There are several advantages of the STL and SCS method [23]. Firstly, it is a straightforward procedure to decompose a time series with missing data. Secondly, it has flexibility in specifying the amount of deviation in seasonal and trend components of time series data. In addition, trend and seasonal components are very robust, so that these are not easily distorted by outliers in the data. Lastly, the STL method is a valuable tool for understanding the complexity of time series data. For SCS plot, it helps visualize patterns both between and within groups during the study period. However, it must be interpreted cautiously because the horizontal lines (average for vertical lines) are highly influenced by large values.

## Conclusions

We found that each province showed slightly different seasonal patterns. Overall, increased incidence rates were observed between July and December during the study period. Our findings provide insight into understanding the seasonal pattern and associated climate risk factors in each province. This study may be another way of providing evidence to aid clinicians when making a diagnosis and foresee the timing of outbreaks, therefore may be utilized to raise public awareness during the high peak seasons in order to prevent or reduce further potential outbreaks or onwards transmission during an outbreak.

## Abbreviations

AR: Autoregressive
CI: Confidence interval
DF: Dengue fever
GEE: Generalized estimating equation
IRR: Incidence rate ratio
MOH: Ministry of health
NBD: Negative binomial distribution
NBR: Negative binomial regression
NDCP: National dengue control programme
NIHE: National institute of hygiene and epidemiology
RMSE: Root-mean-square error
SCS: Seasonal cycle subseries
STL: Seasonal-decomposition procedure based on loess
WHO: World health organization
